# Experiencing quarantine restrictions for adolescents with and without suicidal experience in Russia

**DOI:** 10.1192/j.eurpsy.2021.1567

**Published:** 2021-08-13

**Authors:** D. Dovbysh, M. Ermakova, M. Kiseleva

**Affiliations:** 1 Pedagogy And Medical Psychology, Federal State Autonomous Educational Institution of Higher Education I.M. Sechenov First Moscow State Medical University of the Ministry of Health of the Russian Federation (Sechenov University), Moscow, Russian Federation; 2 Child And Adolescent Psychiatry, Scientific-practical Children’s and Adolescents Mental Health Center n.a. G. Sukhareva, Moscow Department of Health Care, Moscow, Russian Federation

**Keywords:** quarantine, adolescent, Suicide action, COVID-19

## Abstract

**Introduction:**

The life changes and limitations associated with Covid-19 clearly have serious psychological implications. The life of adolescents has also changed significantly in many areas: study, communication with peers, contact with family, etc. and not all adolescents have adapted to these changes equally easily.

**Objectives:**

Study adolescents’ perception of Covid-19, describe the effect of self-isolation on adolescent emotional well-being, and examine changes in the family system through the eyes of a teenager.

**Methods:**

The study involved two groups of adolescents: the first (G1) - 174 students of a Moscow school and the second (G2) - 39 adolescents hospitalized in a children’s psychiatric clinic in connection with suicidal actions. Teenagers filled out the author’s questionnaire, Short Health Anxiety Inventory (Salkovskis), Analysis of Family Anxiety (Eidemiller, Yustickis), Prohibition on the expression of feelings (Kholmogorova).

**Results:**

Participants in G2 significantly more often than G1 reported that their functioning worsened (it became more difficult to study - 72% versus 51%; more difficult to communicate - 76% versus 41%, more conflicts with family members - 49% versus 25%). G2 demonstrated a significantly higher level of family anxiety (M = 17.3 and M = 12.1 p<0.01), a more pronounced prohibition on expressions of negative emotions (M = 37.2 and M = 21.3 p<0.01). The level of anxiety (for one’s own health, well-being of relatives and financial stability) is also significantly higher in G2.

**Conclusions:**

Many adolescents in self-isolation need the support of relatives and the help of specialists. For a number of teenagers, self-isolation has become a crisis situation.

